# Breath-Hold Diving – The Physiology of Diving Deep and Returning

**DOI:** 10.3389/fphys.2021.639377

**Published:** 2021-05-21

**Authors:** Alexander Patrician, Željko Dujić, Boris Spajić, Ivan Drviš, Philip N. Ainslie

**Affiliations:** ^1^Center for Heart, Lung & Vascular Health, University of British Columbia Okanagan, Kelowna, BC, Canada; ^2^Department of Integrative Physiology, University of Split School of Medicine, Split, Croatia; ^3^Faculty of Kinesiology, University of Zagreb, Zagreb, Croatia

**Keywords:** apnea, free-diving, hyperbaric, immersion, diving, nitrogen, breath-holding, spearfishing

## Abstract

Breath-hold diving involves highly integrative physiology and extreme responses to both exercise and asphyxia during progressive elevations in hydrostatic pressure. With astonishing depth records exceeding 100 m, and up to 214 m on a single breath, the human capacity for deep breath-hold diving continues to refute expectations. The physiological challenges and responses occurring during a deep dive highlight the coordinated interplay of oxygen conservation, exercise economy, and hyperbaric management. In this review, the physiology of deep diving is portrayed as it occurs across the phases of a dive: the first 20 m; passive descent; maximal depth; ascent; last 10 m, and surfacing. The acute risks of diving (i.e., pulmonary barotrauma, nitrogen narcosis, and decompression sickness) and the potential long-term medical consequences to breath-hold diving are summarized, and an emphasis on future areas of research of this unique field of physiological adaptation are provided.

## Introduction

Breath-hold diving is a ubiquitous activity for recreation, sustenance, military, and sport; it involves highly integrative physiology and extreme responses to both exercise and asphyxia during progressive elevations in hydrostatic pressure (Ferretti, 2001; Bain et al., 2018b; Fitz-Clarke, 2018). Since the famous anecdote of Giorgios Statti diving to 70 m in 1913; the attainment of 100 m by Jacques Mayol in 1976; and, more recently, Herbert Nitsch reaching a staggering 214 m in 2007, the human capacity for deep breath-hold diving has continually extended the physiological limits and refuted expectations. However, physiological limits do exist and adverse consequences readily occur when these limits are exceeded. The purpose of this review is to outline the basic physics that govern apnea diving, discuss the challenges, physiological responses, and associated clinical consequences of diving to depth.

## Gas Laws

**Figure 1:**
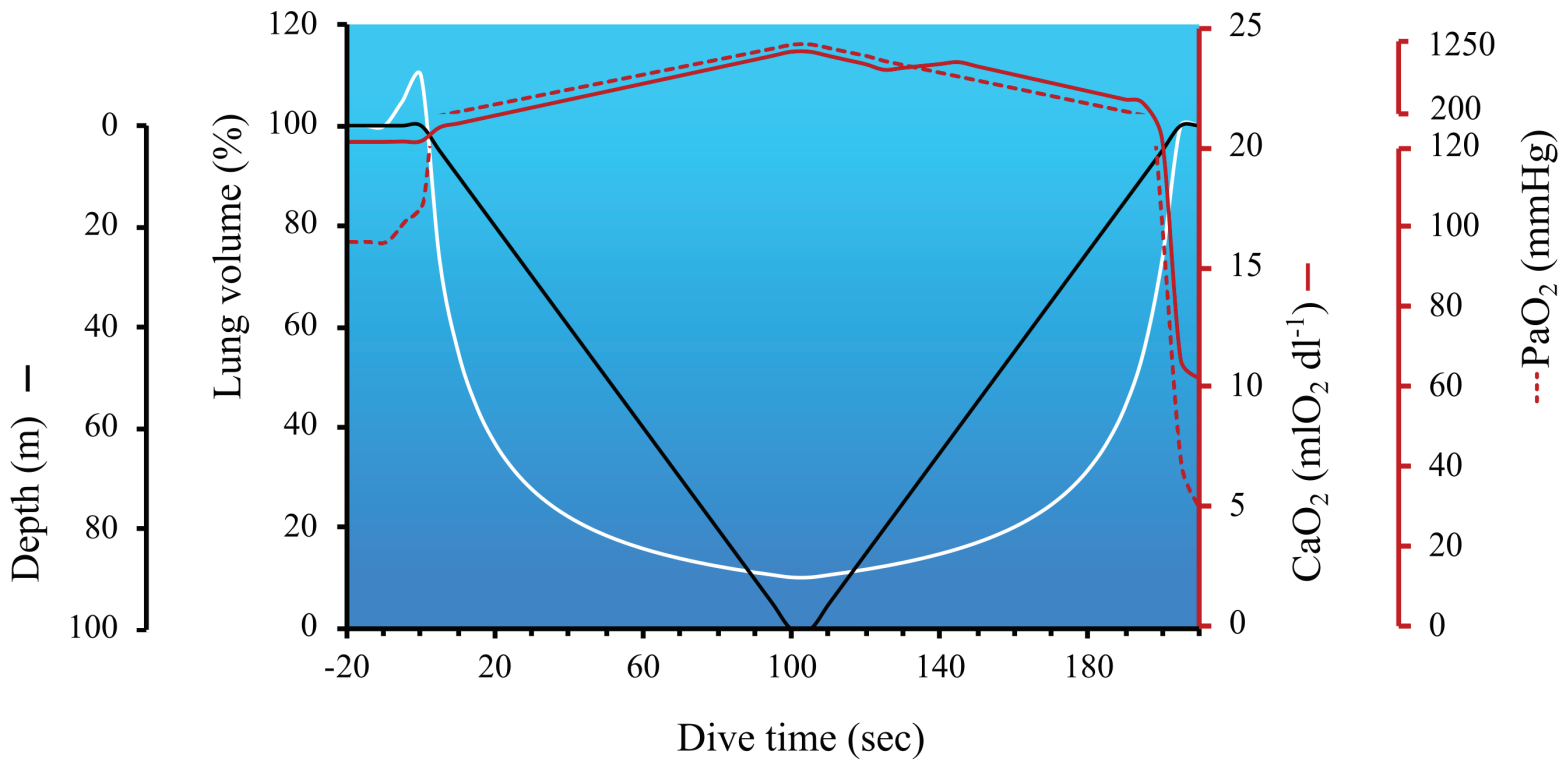
The impact of hydrostatic pressure (also referred to as the absolute pressure in atmospheres; ATA) on lung volume and arterial hypoxia during a simulated dive to 100 m. The hyperbolic nature of lung volume across a dive is due to the nonlinear pressure-volume relationship (calculated in accordance with Boyle’s Law). The temporary increase in lung volume immediately before the start of the dive coincides with lung packing, a maneuver employed by divers to increase the volume of oxygen in the lungs. In this example, with a total dive time of 205 s, the dive speed was set at 1 m/s, with a 5 s bottom time. The arterial oxygen content (CaO_2_) and partial pressure of arterial oxygen (PaO_2_) at the start of the dive were 20.3 mlO_2_ dl^−1^ and 97 mmHg, respectively. Lung packing was performed prior to immersion, thereby increasing lung volume by 10% above TLC, and facilitating a 10 mmHg increase in PaO_2_. CaO_2_ was calculated to be the product of Hb × 1.36(SaO_2_/100) + 0.003(PaO_2_) – and consisted of the following considerations: (1) Hb was assumed to be 15 g dl^−1^, until the early portion of ascent (i.e., ~2 min into dive), when a 5% increase in oxygenated Hb occurred in the circulation (i.e., +0.75 g dl^−1^) *via* splenic contraction. As discussed in *the Ascent* section, splenic contraction is presumed to occur during the latter phase of the dive to coincide with the onset of exercise and growing hypercapnia; (2) SaO_2_ was 98–100% until the last 15 s of the dive when PaO_2_ dropped below 100 mmHg, and SaO_2_ was estimated off a right-shifted O_2_ dissociation curve (Hall et al., 2011); (3) the solubility of O_2_ dl^−1^ of blood (i.e., 0.003) was assumed to remain constant and PaO_2_ was calculated using the following steps – first the partial pressure of alveolar oxygen (P_A_O_2_) was calculated using a modified alveolar gas equation to account for hydrostatic pressure = F_ET_O_2_ (0.1444 during pre/post dive breathing and 0.16 during the dive due to lung packing) multiplied by the product of ATA × (760–47) – (PaCO_2_/R). The partial pressure of arterial carbon dioxide (PaCO_2_) pre-dive was 40 mmHg, and calculated to increase at a rate of 0.06875 mmHg sec^−1^ (derived from PaCO_2_ data during a static apnea in an elite breath-hold diver; Willie et al., 2015 as reviewed in Bain et al., 2018b), resulting in an end-dive PaCO_2_ of 54 mmHg. R was assumed to remain constant at 0.9. ATA increases by 1 every 10 m gain of depth (i.e., 1 ATA on the surface and 11 ATA at 100 m). Blood gases collected at 40 m in breath-hold divers support the notion of hydrostatic-induced hyperoxia – see section *Maximum Depth*; (Bosco et al., 2018). These data also align with predicted P_A_O_2_ across a simulated dive to 150 m (Ferretti, 2001). The metabolic uptake of oxygen during breath-holding was derived from data during static apneas (Willie et al., 2015; Bain et al., 2018b), and assumed to be steady across a dive, at a rate of 0.21205 mmHg sec^−1^. This oxygen uptake was subtracted from P_A_O_2_. PaO_2_ was assumed to mirror P_A_O_2_ until ascent, when an inefficiency of pulmonary gas exchange is expected to occur (Patrician et al., 2021b). In the latter portion of the dive, a widening of the a-A gradient was assumed to be 20 mmHg, which was based on measurements in divers diving beyond 100 m (Patrician et al., 2021a). PaO_2_ within 5 s of surfacing was estimated to be 29.7 mmHg which, in trained breath-hold divers, is slightly above the theoretic limit of consciousness. However, most experienced divers are always conscious of the risk of shallow water blackout (see section: *The last 10 m*).

Humans live in a pressurized air environment, with the pressure at sea level standardized to 760 mmHg or 1 absolute pressure in atmospheres (ATA). Upon diving, pressure increases proportionally with depth, due to the additive weight of the water column. Specifically, for every 10 m (33 ft) of sea water, hydrostatic pressure increases by 1 ATA; therefore, as pressure doubles, the volume of the gas is halved (in accordance with Boyle’s Law which states, in a closed system where temperature remains constant, the volume is directly and inversely proportional to the pressure) and the solubility of all gases increase [in accordance with Henry’s Law which states that the amount of gas absorbed (at the same temperature in liquid) is proportional to the solubility coefficient of the particular gas and their partial pressure]. These concepts are illustrated in Figure 1, which outlines the role of Boyle’s and Henry’s Law on lung volume and circulating oxygen levels across a dive to 100 m.

## Phases of a Dive

To demonstrate the integrated nature of breath-hold diving to depth in humans, the physiological responses are presented as they occur during a dive and upon resurfacing.

### The First ~20 Meters

Even before the dive, while floating at the surface, physiological changes are already occurring due to the partial state of immersion – consisting of fluid shifts, regional blood flow redistribution, altered cardiopulmonary hemodynamics, and autonomic activity (reviewed in; Pendergast et al., 2015). During the final inspiration, many divers perform glossopharyngeal insufflation (also known as lung packing). Lung packing has been shown to increase lung volume by 11–26% (up to +3 L; Lindholm and Nyrén, 2005; Loring et al., 2007; Chung et al., 2010; Walterspacher et al., 2011; Mijacika et al., 2017; Patrician et al., 2021a); however, too much packing can increase the risk of lung barotrauma (Chung et al., 2010) or pre-dive syncope, as lung hyperinflation alters cardiac mechanics (decreases end-systolic and diastolic volumes), reduces cardiac output (Mijacika et al., 2017; Kjeld et al., 2021), and facilitates cerebral hypoperfusion (Van Lieshout et al., 2003).

#### Mammalian Diving Response

To facilitate a reduction in oxygen consumption, the mammalian diving response initiates a vagally mediated increase in parasympathetic nerve activity that reduces heart rate (i.e., bradycardia; Olsen et al., 1962b; Craig, 1963) and sympathetically mediated vasoconstriction of peripheral vascular beds (i.e., transient reduction in blood flow to non-essential organs; Brick, 1966; Heistad et al., 1968). Both of these responses are augmented with facial immersion, especially with cool water (Hurwitz and Furedy, 1986; Schuitema and Holm, 1988; Schagatay and Holm, 1996). Even though there remains some ambiguity regarding quantifying the degree of bradycardia (i.e., when selecting the pre-dive baseline heart rate), heart rates have been reported to decrease to as low as 20–30 beats per minute (Ferrigno et al., 1997; Ferretti, 2001; Bain et al., 2018b), or even lower (Arnold, 1985).

#### Cardiac Dyssynchrony

An interesting, albeit less common phenomenon exhibited by divers is the presentation of cardiac arrhythmias (or dyssynchrony) during a dive. Such dyssynchrony has been demonstrated in both freely diving humans equipped with waterproof electrocardiographic (ECG) units (Olsen et al., 1962b; Scholander et al., 1962; Ferrigno et al., 1991; Hansel et al., 2009; Patrician et al., 2021a), including the Ama (Sasamoto, 1965; Hong and Rahn, 1967), and under experimental hyperbaric conditions (Ferrigno et al., 1997). The implications of such dyssynchrony are not clear, especially whether they infer a malignant or benign disposition. The mechanisms driving these dyssynchronous ECG signals are also not established, but likely reflect marked “autonomic conflict” between parasympathetically mediated bradycardia (i.e., diving response) and sympathetically driven tachycardia (i.e., cold shock and/or exercise response; Shattock and Tipton, 2012). Whether phenotypic predisposition is required or simply contributory in its genesis, the hydrostatically induced centralization of blood volume from the legs to thorax (Miyamoto et al., 2014), and concomitant mechanical release of hyperinflation-induced compression of the heart from lung packing (Mijacika et al., 2017) could alter baroreflex control (Chen et al., 2006) and chronotropic drive due to cardiac enlargement and activation of right atrial stretch receptors. Ultimately, further investigation and importantly the potential association with elevated myocardial infarction risk, has yet to be elucidated.

### Passive Descent

Within the first 20 or so meters, the diver reaches a point of neutral buoyancy. In reality, however, the exact depth of neutral buoyancy depends on a variety of factors (e.g., body composition, neck/hip weights, wetsuit composition/thickness, surface lung volume, water density/salinity). At this depth, the weight of the water column exerted upon the diver equals the buoyancy of the diver – meaning the diver will not float or sink. Beyond this point, however, the hydrostatic force overcomes the buoyancy of the diver, and the diver becomes negatively buoyant and enters a state of continuous free-fall. At this stage (or earlier), peripheral vasoconstriction is likely maximized *via* elevations in sympathetic nervous system activity, which limits distribution of blood to non-essential organs (e.g., kidney and skeletal muscles; Fagius and Sundlöf, 1986; Heusser et al., 2009; Kyhl et al., 2016). This re-distribution has been well demonstrated in Weddell seals, where overwhelming vasoconstriction is evident in all organs, except in the brain (Zapol et al., 1979). In humans performing dry or facial immersion static apnea, the combined influence of (1) sympathetic excitation and chemoreflex engagement from combined inputs of hypoxia, hypercapnia, lack of ventilatory inhibition (Heusser et al., 2009; Steinback et al., 2010) and (2) peripheral vasoconstriction, likely drive the increases in mean arterial pressure; this has been measured to increase to 150 mmHg (Breskovic et al., 2011; Bain et al., 2015), and even reach 200 mmHg (during apnea at rest and with exercise; Bjertnaes et al., 1984; Ferrigno et al., 1997; Perini et al., 2010). However, blood pressure data during underwater diving are scarce. In an innovative study by Ferrigno et al. (1997), two participants with radial artery catheterization performed breath-holds in a hyperbaric chamber to simulate the hydrostatic load of a dive to 50 m. While there were substantial elevations in mean arterial pressure during descent (with occasional systolic peaks in one diver reaching 345 mmHg), mean arterial pressure appeared to slightly subside or even decrease during the latter portion of the dive (Ferrigno et al., 1997). These blood pressure responses in humans contrast to those in marine mammals, who demonstrate relatively stable blood pressure across a dive (Irving et al., 1942; Ponganis et al., 2006) due to an enhanced mammalian diving response (e.g., extreme bradycardia to 2–3 bpm; Williams et al., 2017; Goldbogen et al., 2019) and anatomical adaptations (e.g., hepatic sinus and venous vasculature; Harrison and Tomlinson, 1956; Ponganis, 2011).

#### Lung Compression

In accordance with Boyle’s Law, and illustrated in Figure 1, lung volume decreases with increasing depth. Assuming the diver fully inspires at the surface, the hydrostatic-induced reduction in lung volume would reduce lung volume to approximately residual volume (RV) by 40–50 m. The RV-equivalent depth has certainly been theorized to represent a hurdle in deep diving, but since at least the 1960s, divers have successfully attained progressively deeper records (Fitz-Clarke, 2018). However, in studies of humans diving during both simulated and sea conditions, the RV-equivalent depth may still constitute a physiological threshold of lung injury risk (Lindholm and Lundgren, 2009; Patrician et al., 2021b). The risk of lung injury could be exaggerated when divers perform large thoracic movements at depth (e.g., an exaggerated swimming stroke), because the coincidingly elevated alveolar pressures proportionally dictate the degree of tissue strain (Hooke’s Law). If severe enough, this tissue strain could overcome the structural capacity of lung tissue and contribute to capillary stress failure and pulmonary edema (see section: *Risk of lung squeeze*). However, even in the absence of overt barotrauma, divers diving to – or below – RV-equivalent depths, have been shown to present with transient impairments in pulmonary gas efficiency and subtle alterations in lung compliance at ~2.5 h post-dive (Patrician et al., 2021b).

The mechanics and geometry of hydrostatically induced lung compression are complex and likely determined by a number of factors. First, the compositional organization of collagen, elastin, and other lung tissue constituents in the lungs can dictate the mechanical behavior of tension and compression (Andrikakou et al., 2016). Humans lack many of the thoracic and lung adaptations found in diving mammals, which are designed to manage lung compression/collapse and reopening (reviewed in; Ponganis et al., 2003; Castellini, 2012). Second, airway modeling of head-up descent in humans (e.g., when using an underwater sled) suggests that airway collapse occurs early in descent (with basal segments collapsing as early as 18 m), and collapse occurs in a heterogeneric pattern (Fitz-Clarke, 2007). Third, there is evidence that the active centralization of blood into the thorax – referred to as the “blood shift” – alleviates the reduction in gas volume, and therefore provides some protection for the lungs against collapse. Using a non-invasive approach to estimate changes in regional blood volume (impedance plethysmograph), Schaefer and colleagues estimated that 850 ml of had translocated into the thorax in a diver diving to 40 m (Schaefer et al., 1968). Likewise, a 1.4–1.7 L increase in thoracoabdominal volume has also been reported in three divers performing simulated dives to 45–55 m (*via* wet hyperbaric chamber; Ferrigno and Lundgren, 2003). Given that head-out immersion induces translocation of blood to the thorax, such thoracic centralization during diving is not an unreasonable notion (reviewed in; Pendergast et al., 2015), and has long since been postulated to contribute to divers being capable of diving beyond RV, without ill-consequence (Craig, 1968).

Despite the numerous dangers of extreme diving, a recent report of two world-champion breath-hold divers, diving to 102 and 117 m, highlight the incredible tolerability of highly adapted individuals to extreme levels of hydrostatic-induced lung compression (Patrician et al., 2021a). These observations are consistent with the accomplishments of many highly trained divers who have performed dives to extreme depths (100–214 m), despite profound reductions in lung volumes [between 9 and 4.5% of surface volume (calculated in accordance with Boyle’s Law), with modeling suggesting 55–85% of total airway collapse, respectively Fitz-Clarke (2007)].

### Maximum Depth

Upon reaching maximum depth, the elevated hydrostatic pressure facilitates a peak in the partial pressure of arterial oxygen (PaO_2_), due to the hyperbaric-induced increase in gas partial pressure and solubility (illustrated in Figure 1). In a recent study conducted at the “Y-40 THE DEEP JOY” pool in Italy, arterial blood gases were evaluated at a depth of 40 m in six breath-hold divers (Bosco et al., 2018). In four of the six divers, PaO_2_ increased from 94 ± 6 mmHg on the surface to 263 ± 32 mmHg at 40 m. Intriguingly, two of the divers did not demonstrate the expected hyperoxia at depth (PaO_2_ at 40 m was 68 ± 10 mmHg). The authors postulated that ventilation perfusion mismatching and right-left intrapulmonary shunt, due to atelectasis (i.e., airway closure) gave rise to these divergent findings in these two divers.

#### Nitrogen Narcosis

Depending on bottom depth and time at depth, the partial pressure of alveolar nitrogen (P_A_N_2_) will be inordinately elevated, and facilitate diffusion of nitrogen into the blood. Early work in diving mammals (Kooyman et al., 1972) and humans (Radermacher et al., 1992) have demonstrated that the nitrogen tension in the blood rises during breath-hold diving. Even in the normal upright lung, nitrogen uptake has been demonstrated to occur during rest (Canfield and Rahn, 1957; West, 1962). In fact, nitrogen narcosis is not an uncommon feature in breath-hold dives exceeding ~70–90 m, with transient amnesia being a prevalent symptom. In a recent report by Patrician et al. (2021a), two divers spent 26 and 42 s, respectively, beyond 90 m – with maximum depths of 102 and 117 m. As P_A_N_2_ (and therefore PaN_2_) increases with depth, the total time exposed at these extreme depths evidently appears to play a crucial role in both divers reporting narcosis. However, despite its prevalence being higher than reported (potentially due to amnesia; Ferrigno and Lundgren, 2003), there are only a few published examples in the literature (Lindholm and Lundgren, 2009; Fitz-Clarke, 2018; Patrician et al., 2021a). Ultimately, the impact and physiological influence of nitrogen narcosis, and contributory role in pathogenesis of decompression sickness in breath-hold divers has not been thoroughly studied, and its awareness is important to reduce the risk for adverse incidents.

### Ascent

To ascend, the diver must propel themselves toward the surface and overcome the negative buoyancy and exaggerated drag. With peripheral vasoconstriction already established, muscular contraction will likely rely heavily on anaerobic sources, and lead to an increase in lactate production. While early reports suggested that blood lactate only mildly increased following apneic exercise [i.e. foot-pedalling during apneas of 104 s in a hyperbaric chamber; Olsen et al., 1962a)], more recent assessments in divers performing horizontal underwater swimming (to 173 ± 25 m and 135 ± 23 m, either with and without using fins, respectively) have reported blood lactate concentrations of 10 ± 2mmol L^−1^ within 2–4 min of surfacing (Schagatay, 2010). Interestingly, from a comparative perspective, Weddell seals do not demonstrate a rise in lactate concentration until dives exceed 20 min (Kooyman et al., 1980); and lactate concentrations only reach comparable human values once dives exceed +40 min (Kooyman and Ponganis, 1998).

Depending on the dive duration, divers can experience involuntary breathing movements (IBMs). These IBMs are essentially diaphragmatic contractions, which signify the “physiologic break-point” and ensuing struggle phase (Schagatay, 2009; Bain et al., 2018b). The intensity of IBMs dramatically increase, coinciding with an amplifying drive to breathe, progressive hypoxemia, hypercapnia, and hypertension. Interestingly, IBMs have been shown to help transiently alleviate the restrictions on cardiac output, especially at higher lung volumes (Palada et al., 2008) and improve cerebral oxygenation (Dujic et al., 2009). However, as lung compression alleviates with ascent, the onset of IBMs may conversely introduce exaggerated intrathoracic and transpulmonary pressure fluctuations. If such abrupt fluctuations in thoracic pressure were to occur below a diver’s RV-equivalent depth, and/or coincided with instances of abrupt alveolar reopening (postulated to occur during ascent), the associated tissue strain (in accordance with Hooke’s Law) could lead to pulmonary barotrauma/lung squeeze. In fact, hemoptysis following a dive, has been attributed to IBMs (Kiyan et al., 2001). The notion of abrupt alveolar reopenening is derived from modeling work by Fitz-Clarke (2007). This work suggests, that during ascent, disproportionate coordination between transpulmonary pressure and regional airway surface tension can arise in alveolar units that have collapsed. When the airway surface tension of a collapsed segment is eventually overcome, the widened transpulmonary pressure gradient leads to an abrupt compensatory airway equalization [referred to as airway/alveolar “popping”; Fitz-Clarke (2007)]. Ultimately, the physiological implication(s) of IBMs or alveolar “popping” on pulmonary barotrauma has yet to be elucidated.

#### Spleen Contraction

The spleen has for centuries been considered to act as a dynamic reservoir, capable of expelling blood into the circulation, with a hematocrit concentration higher than arterial blood (Barcroft and Poole, 1927) – intrasplenic hematocrit estimates of 64–78% in mice, 75% cats and 90% in dogs (Opdyke and Apostolico, 1966). In humans, a 18–35% decrease in spleen volume has led to an increase in hematocrit by 2–6% during breath-holding (Schagatay et al., 2001; Stewart and McKenzie, 2002; Baković et al., 2003; Schagatay et al., 2012). Interestingly, the Ama (an indigenous diving population) have demonstrated an increase in hematocrit by 10.5% following 1 h of repetitive diving to 5–7 m (Hurford et al., 1990). Certainly, such a splenic contraction may coincide with peripheral vasoconstriction and sympathetic activation during descent, as splenic contraction has been demonstrated to occur rapidly (within 3 s of apnea onset; Palada et al., 2007). However, the combined aspects of exertion and hypercapnia (Richardson et al., 2012), or developing hypoxemia during the last part of ascent, splenic contraction would certainly be timely during this phase of the dive. Ultimately, irrespective of when splenic contraction occurs during a dive, based on the calculations in Figure 1, the hemoconcentration *via* splenic contraction would increase CaO_2_ (assuming all else remains equal) by ~5%, which may proffer a protective benefit immediately upon surfacing, to avoid hypoxic loss of consciousness.

### The Last 10 Meters

The risk of shallow water blackout and hypoxic syncope is notoriously high within the last 10 m of ascent. Modeling conducted by Ferretti (2001) has estimated P_A_O_2_ of a diver returning from a dive at 150 m, to be as low as 25 mmHg, which lies close to the theoretic limit of consciousness – postulated to be when PaO_2_ falls below 20 mmHg (Ernsting, 1963). This modeling aligns with Figure 1 (where PaO_2_ upon surfacing from a 100 m dive was estimated to be 29.7 mmHg), and is supported by static studies in trained breath-hold divers. For example, Lindholm and Lundgren (2006) showed that post-apnea P_ET_O_2_ values of 20.3 mmHg (range: 19.6–21.0 mmHg) coincided with loss of motor control, whereas P_ET_O_2_ values of only ~3 mmHg higher (mean 23.0 mmHg; range 22.4–23.6 mmHg) did not (Lindholm and Lundgren, 2006). Additionally, in elite apneists under “dry” laboratory conditions, a series of studies utilizing radial artery (Willie et al., 2015) and jugular venous catheterizations (Bain et al., 2016, 2017, 2018a) have demonstrated end-apnea PO_2_’s of 29.6 ± 6.6 mmHg and 25 ± 6 mmHg, respectively (including an extreme end-apnea PaO_2_ of 23 mmHg following a 435 s breath-hold; Bailey et al., 2017), without syncope. Comparatively, PaO_2_ in freely diving elephant seals has been measured as low as 12 mmHg (corresponding to an SaO_2_ of only 8%) just prior to surfacing (Meir et al., 2009).

During severe hypoxemia, there is a reduction in oxidative metabolism of the brain (Bain et al., 2017), which complement the gradual rise in cerebral blood flow that occurs across an apnea, serving to sustain cerebral oxygen demands (Willie et al., 2015) – and ultimately prolong consciousness. It appears that diving (versus terrestrial) mammals have distinct neural adaptations (i.e., a different distribution of neuroglobins that are found in higher concentrations in glial cells/astrocytes than in neurons) which predispose tolerance to hypoxia and resistance to reactive oxygen species (Folkow et al., 2008; Mitz et al., 2009), and also have been found to have an enhanced tolerance to lactate and changes to exogenous substrate availability (Czech-Damal et al., 2014). For example, in early studies with restrained Weddell seals performing maximal effort apneas, surrogate indexes cerebral metabolism (i.e., abnormal electroencephalogram slow waves) suggest impairments only occur when arterial and cerebral venous PO_2_ drop to 10 mmHg and 2.6 mmHg (Elsner et al., 1970; Kerem and Elsner, 1973).

### Surface Protocol

If this was a competitive dive, the dive would only be considered successful by judges [from either the Association Internationale pour le Développement de l’Apnée (AIDA) or the World Confederation of Underwater Activities (CMAS)], if the diver demonstrated control upon surfacing. For example, AIDA requires divers to remove their facial equipment (i.e., goggles and nose clip), perform an “OK” hand-signal and verbally state “*I am OK*,” in that order, all the while continuously maintaining airways above the water. Given risk of syncope (see section: *Last 10 m*), divers employ techniques upon surfacing to enhance cerebral perfusion (Fernández et al., 2019). However, we cannot stress with enough strength, or more strongly recommend against extreme diving, given the profound risk of drowning. Even though competitions employ vast safety protocols (including safety divers, winch and video/sonar systems) to ensure diver safety, recreational incidents are unfortunately on the rise – with 52 fatalities in 2017 and the most injuries since 2004 (DeWitt et al., 2019).

#### Risk of Lung Squeeze

With a delicate pulmonary capillary interface (West et al., 1991), it is perhaps not surprising that lung injury can occur, due to the cumulative forces of hydrostatic-induced compression and decompression of both the lungs and thoracic cage, centralization of blood volume, hypertension, exertion, and hypercapnic hypoxia. Such an injury, commonly referred to as lung squeeze, is a form of pulmonary barotrauma that has been extensively reviewed (Ferrigno and Lundgren, 2003; Lindholm and Lundgren, 2009; Dujic and Breskovic, 2012; Mijacika and Dujic, 2016; Moon et al., 2016; Kumar and Thompson, 2019; Schipke et al., 2019). Lung squeeze manifests shortly after surfacing and is characterized by pulmonary edema and hemoptysis (Boussuges et al., 1999; Lindholm et al., 2008; Patrician et al., 2021a), and is often associated with productive cough, dyspnea, and chest tightness (Cialoni et al., 2012); decrements in lung function and reduced oxygen saturation (Linér and Johan, 2008); and an impairment in pulmonary gas efficiency (Patrician et al., 2021a). Certainly, any injury to the delicate pulmonary capillary interface is serious and requires the appropriate medical attention. But due to the elevated risk of drowning and/or meager accessibility to medical aid, it is potentially shortly upon surfacing when the consequences of barotrauma can be the most consequential, or even fatal (Vestin, 2015). The exact phase of a dive when the integrity of the pulmonary capillaries become compromised – and exact mechanisms involved – has been explored since the late 1950’s (Carey et al., 1956; Craig, 1968; Ferretti, 2001; Fitz-Clarke, 2007; Lindholm and Lundgren, 2009; Ferretti et al., 2012). Yet, it is still not clear whether lung squeeze can be attributed to the compression and alveolar collapse during descent, the cumulative strain and capillary stress failure under compression, or the de-compression and alveolar reopening during ascent.

An important future direction that remains to be addressed in breath-hold divers, is the development of appropriate recovery strategies for breath-hold divers who suffer from a single bout or repetitive incidents of pulmonary barotrauma or lung squeeze. Abstinence from diving and rest is certainly a necessity, as the risk of swimming-induced pulmonary edema reoccurrence has been reported to be as high as 75% (reviewed in; Grünig et al., 2017). However, the nature and duration of such rest is unclear. The only available literature on future barotrauma risk is limited to SCUBA (self-contained underwater breathing apparatus) divers, and the findings are inconclusive (Calder, 1985; Russi, 1998).

#### Risk of Decompression Sickness

Decompression sickness (DCS) or “Taravana” can occur in breath-hold divers, especially those diving repetitively (e.g., spearfishing, safety divers, and/or use of underwater scooters) and those performing extreme depths (reviewed in; Cross, 1965; Paulev, 1965; Rahn and Yokoyama, 1965; Schipke et al., 2006; Fitz-Clarke, 2009; Lemaitre et al., 2009; Moon and Gray, 2010; Dujic and Breskovic, 2012). The pathology of DCS, including its manifestation and risk factors have been extensively reviewed elsewhere (Brubakk and Neuman, 2003); however, in breath-hold divers, symptoms can range from dizziness, nausea, thoracic/skin/joint pain, hemiplegia, paresis, dysarthria, vertigo, and unconsciousness, with short- to long-term prognoses (Cross, 1965; Schipke et al., 2006; Cortegiani et al., 2013; Tetzlaff et al., 2017). The consequences of DCS are related to the affinity of certain tissues and the rate at which they uptake nitrogen. For example, the brain, heart, and viscera saturate within a couple of minutes, whereas in fat tissues, nitrogen continuously rises (Schipke et al., 2006). Likewise, upon ascent, the half-life of nitrogen is less in neural tissues, followed by skin and muscle, and then joints, ligaments, and bones (Rusoke-Dierich, 2018). Even though the rapid uptake of nitrogen occurring in neural tissue and blood may only manifest as narcosis, the rate of nitrogen clearance is a serious concern for divers, since ascent cannot be feasibly or safely slowed. It is perhaps not surprising that intravascular bubbles have been reported following spearfishing, where short surface times and the repetitive dives occur with often insufficient times to allow nitrogen clearance (Cialoni et al., 2016). Although this observation is not a universal finding (Boussuges et al., 1997; Gargne et al., 2012), arterial gas embolism following breath-hold diving has been implicated in stroke risk (Tetzlaff et al., 2017). Ultimately, modeling has suggested that the risk of decompression sickness up to 100 meters is quite low, but increases non-linearly to 5–7% at 230 m, where total lung collapse is anticipated to occur (Fitz-Clarke, 2009). This aligns with reports of nitrogen narcosis in elite divers diving beyond 100 m (Patrician et al., 2021a).

## Are There Long-Term Medical Consequences to Extreme Breath-Hold Diving?

There are indigenous diving populations, such as the Ama in Japan, the Haeyeno in Korea (Teruoka, 1932; Rahn and Yokoyama, 1965; Hong and Rahn, 1967; Vaneechoutte et al., 2011), and the Bajau in Indonesia (Abrahamsson and Schagatay, 2014; Ilardo et al., 2018) who still practice breath-hold diving as their primarily means of harvesting food. Early reports from the Ama seemed to limit occupational aliments to the ear, nose, and throat (e.g., hearing loss, stenosis of Eustachian tube, sinusitis; Harashima and Iwasaki, 1965). Oh and colleagues provide recent evidence to indicate an elevated prevalence of chronic kidney disease in Korean Haenyeo, based on a large cohort of Korean Haenyeo subsistence divers (*n* = 715) and controls (*n* = 715; matched for a variety of covariates, such as age, hypertension, cardiovascular disease, and circulatory parameters; Oh et al., 2017). However, the nature of this sustenance diving starkly contrasts the profound degrees of hypoxemia, hyperoxia, and hypercapnia regularly experienced by modern competitive free-divers and spear-fishers who push their physiological limits. Therefore, whether chronic/lifelong extreme diving with profound exposures incurs any long-term consequences is an important future research topic.

### Brain Biomarkers and Cognitive Function

It is perhaps not entirely surprising that increases in blood-brain barrier and astrocyte damage markers (i.e., S100β) have been shown immediately following maximal static apneas (Andersson et al., 2009), which can persist for >1 day following a blackout (Linér and Andersson, 2009). An elevated oxidative state following sustained diving (Theunissen et al., 2013) increased cortisol, copeptin, brain natriuretic peptide, and ischemia-modified albumin (Marlinge et al., 2019). However, in another study, there was an increase in another brain neuronal damage marker (i.e., neuron-specific enolase), yet without changes in other related brain and cardiac markers (Kjeld et al., 2015). At least with static apneas without blackout, the rise in some brain damage markers that suggest potential disruption of the blood-brain barrier seem to occur in the absence of neuronal-parenchymal damage (Bain et al., 2018a). However, with supramaximal performances during static and dynamic disciplines – which have moderate incidences of blackout – the risk is likely higher. With regards to cognitive function, however, the findings are mixed – with some reports suggesting normative scoring on neuropsychological tests (Ridgway and McFarland, 2006) and others showing slight decrements which were correlated with maximum static apnea duration (Billaut et al., 2018). More cross-sectional and longitudinal research is required on this topic.

### Lung Function

Another relevant question related to the long-term consequences of apnea diving pertains to the lungs, and in particular, the small airways. To enhance deep diving performance, divers regularly perform (1) passive thoracic stretches (e.g., static movements to elicit varying degrees of torsion and tension within the thorax, at elevated and minimal lung volumes) and (2) active lung stretches (*via* inspiratory and expiratory glossopharyngeal breathing, to alter lung volume >TLC and <RV respectively; Lindholm et al., 2009). These techniques aim to increase TLC and improve tolerance to hydrostatic-induced lung compression (i.e., to depths beyond RV-equivalent depth). However, any increase in lung compliance could come at the cost of exaggerated elastance and a premature closing pressure. Therefore, it is logical to hypothesize that the lungs of elite divers could become pendulous over time. Unfortunately, there is a scarcity of longitudinal data on lung function in breath-hold divers. However, some unique data exist in four male trained divers who were longitudinally tracked over 3 years (personal best depths ranging between 32 and 64 m; Walterspacher et al., 2011), and one well-trained male diver over 8 years (personal best depth of 88 m; Seccombe et al., 2013). Although it was concluded that there was not clear evidence of any long-term deleterious effects of diving or training on the lungs, elevations in TLC coincided with reductions in FEV_1_/VC; however, these data should be confirmed in a lager sample size incorporating more sophisticated measures of pulmonary function. Cross-sectional reports indicate that the FEV_1_/FVC ratio, at least in mostly recreational diving cohorts, does not appear markedly different from matched controls (0.79 for both groups; Ferretti et al., 2012), or is even improved (0.94 in elite divers vs. 0.84 in controls; Lemaître et al., 2010). However, mean FEV_1_/FVC ratios of (1) 0.75 was demonstrated in competitive divers with a history of performing lung packing (Tetzlaff et al., 2008), (2) 0.74 was demonstrated in actively training and competing divers (Walterspacher et al., 2011), and (3) 0.76 was demonstrated in recreational to elite trained divers (Patrician et al., 2021b), where the highest trained divers (*n* = 8) had a mean FEV_1_/FVC ratio of 0.71; and (4) 0.67–0.69 was demonstrated in the longitudinally tracked diver, after 5–8 years of diving/training (Seccombe et al., 2013). Certainly, FEV_1_/FVC provides limited insight into lung function and most of these data do not conform to the classical criteria for obstructive impairment (i.e., <0.70%). Therefore, additional metrics need to be evaluated, especially given the difficulty in evaluating pre-clinical small airway disease. In the longitudinal study by Walterspacher et al. (2011), mean static and dynamic lung compliance was unaltered after the 3-year study, however the cohort was divided, with equal proportions of divers demonstrating an increase or decrease in compliance. In a more recent study by Patrician et al. (2021b) surrogate measurements of lung compliance (i.e., airway resistance and reactance *via* forced oscillation technique) appears mildly altered within 170 min post-dive, but were not outside the normative ranges at either baseline or post-dive. Ultimately, large scale longitudinal studies (with bronchodilator reversibility tests) are essential to support or refute the notion that diving – or the aggressive training strategies typically employed by divers to aid performance (e.g., inspiratory and expiratory glossopharyngeal breathing) – might incur any risk of pulmonary function decline.

## Conclusion

Ever since the early diving studies in the Japanese Ama (Teruoka, 1932), our scientific exploration of human breath-holding capacity has advanced our understanding of human physiology and adaptation. In this review, we have discussed the physiological responses that occur across the phases of a dive, and highlighted the challenges driven by hyperbaria. Future longitudinal and cross-sectional studies are needed to fully elucidate the potential clinical consequences of apnea and diving.

## Author Contributions

All authors contributed to the conception and design of the work. AP drafted the manuscript. All authors revised the manuscript critically for intellectual content, approved the final version, and agreed to be accountable for all aspects of the work.

### Conflict of Interest

The authors declare that the research was conducted in the absence of any commercial or financial relationships that could be construed as a potential conflict of interest.
